# Education plays a greater role than age in cognitive test performance among participants of the Brazilian Longitudinal Study of Adult Health (ELSA-Brasil)

**DOI:** 10.1186/s12883-015-0454-6

**Published:** 2015-10-09

**Authors:** Valéria Maria de Azeredo Passos, Luana Giatti, Isabela Bensenor, Henning Tiemeier, M. Arfan Ikram, Roberta Carvalho de Figueiredo, Dora Chor, Maria Inês Schmidt, Sandhi Maria Barreto

**Affiliations:** Faculty of Medicine, Universidade Federal de Minas Gerais, Belo Horizonte, Brazil; Faculty of Nutrition, Universidade Federal de Ouro Preto, Ouro Preto, Brazil; Faculty of Medicine, Universidade de São Paulo, São Paulo, Brazil; Department of Epidemiology, Erasmus Medical Center, Rotterdam, Netherlands; Campus Centro Oeste Dona Lindu, Universidade Federal São João del Rey, Belo Horizonte, Brazil; Public Health School, Fundação Oswaldo Cruz, Rio de Janeiro, Brazil; Faculty of Medicine, Universidade Federal do Rio Grande do Sul, Porto Alegre, Brazil; ELSA Research Center, Hospital Borges da Costa, Universidade Federal de Minas Gerais, Av. Alfredo Balena 100, 30130-100 Belo Horizonte, Brazil

**Keywords:** Cognition, Socio-demographics, Cohort study, Neuropsychological tests

## Abstract

**Background:**

Brazil has gone through fast demographic, epidemiologic and nutritional transitions and, despite recent improvements in wealth distribution, continues to present a high level of social and economic inequality. The ELSA–Brasil, a cohort study, aimed at investigating cardiovascular diseases and diabetes, offers a great opportunity to assess cognitive decline in this aging population through time-sequential analyses drawn from the same battery of tests over time. The purpose of this study is to analyze the influence of sex, age and education on cognitive tests performance of the participants at baseline.

**Methods:**

Analyses pertain to 14,594 participants with aged 35 to 74 years, who were functionally independent and had no history of stroke or use of neuroleptics, anticonvulsants, cholinesterase inhibitors or antiparkinsonian agents. Mean age was 52.0 ± 9.0 years and 54.2 % of participants were women. Cognitive tests included the word memory tests (retention, recall and recognition), verbal fluency tests (VFT, animals and letter F) and Trail Making Test B. Multivariable linear regression analysis was used to determine the influence of sociodemographic characteristics on the distribution of the final score of each test.

**Results:**

Women had significant and slightly higher scores than men in all memory tests and VFT, but took more time to perform Trail B. Reduced performance in all tests was seen with an increase age and, more importantly, with decrease level of education. The word list and VFT scores decreased at about one word for every 10 years of age; whereas higher-educated participants scored four words more on the word list test, and six or seven more correct words on VFT, when compared to lower-educated participants. Additionally, the oldest and less educated participants showed significant lower response rates in all tests.

**Conclusions:**

The higher influence of education than age in this Brazilian population reinforce the need for caution in analyzing and diagnosing cognitive impairments based on traditional cognitive tests and the importance of searching for education-free cognitive tests, especially in low and middle-income countries.

## Background

Dementia is an emerging public health issue worldwide, including in low and middle-income countries. Brazil is an emerging middle-income country with a rapidly aging population. Yet, environmental and social structures are still inadequate to face the challenges of this demographic shift. Despite recent improvements in wealth distribution, there are marked social inequalities and economic disparities among Brazilians, especially among the middle-aged and the elderly. The income distribution in Brazil is still one of the worst in the world and illiteracy and low schooling level are quite frequent among the population aged 50 years and above. In 1940, 56 % of the Brazilian population were illiterate, a percentage which declined to 40 % in 1960 and to 13.6 % in the year 2000 [1]. These differences have a major impact on the distribution of illness and mortality burden in the population [2]. With regard to dementia, an important implication of this inequality is that a correct interpretation of cognitive test performance remains difficult, as the majority of cognitive tests was conceived and tested in more educated and developed countries.

Even though functional impairment is required for a diagnosis of dementia, longitudinal analyses of an age-related cognitive decline rely mainly upon psychometrics procedures that are strongly dependent on educational background [3–5].

As such, this approach may result in misclassification of less-educated individuals, who could be labeled as having a cognitive deficit. A study on the effects of education and culture on the validity of the Geriatric Mental State examination, a widely used comprehensive mental health research assessment for older persons, found that, in spite of its high sensitivity, in some places of India one-half to two-thirds of the least educated individuals were misdiagnosed. In Brazil, Venezuela, Argentina, Chile and Mexico, almost half of the dementia cases were misdiagnosed [6].

The ELSA-Brasil, an ongoing cohort study comprising 15,105 adult participants, was designed to evaluate the incidence of and prognostic factors for cardiovascular diseases and diabetes [7], and includes cognitive testing with time-sequential analyses drawn from the same battery of tests over time [8]. As profound socioeconomic changes have taken place in the last decades in Brazil, especially in terms of educational achievement, these effects can be captured for ELSA participants’ birth cohorts with different social and educational backgrounds.

Cognition of the study’s participants was evaluated by a core battery of tests used to assess memory, language, visuospatial and executive functions. These tests are widely used in clinical and epidemiological studies among adults living in developed countries and were validated to the Brazilian elderly [9, 10]. However, generalization to different populations may be limited or even inadequate [11]. The cut-offs values used to classify mild cognitive impairment and dementia were derived from the Brazilian elderly and, therefore, may not apply to studies with younger populations [11, 12].

Although it is well known that age, education and intelligence affect cognitive tests performance, we assume that the magnitude of this influence will vary depending on the socio-demographic composition of each population.

This articles aims to analyze the influences of sex, age and education on the performance on various cognitive tests among civil servants of six Brazilian cities in this large cohort of adults from different regions of Brazil.

## Methods

### Study design

ELSA-Brasil is a cohort of 15,105 civil servants from universities and a research institutes located in six Capitals from three different regions of Brazil: Northeast, Southeast and South. The cohort includes volunteers (76 % of the final sample) and actively recruited participants (24 %) from listings of civil servants. Efforts were made to recruit similar proportions of men and women, as well as predefined proportions of age groups and occupational categories. The strategy to recruit civil servants was motivated to ensure a minimal dropout rate during follow up. All participants were Portuguese speakers, functionally independent and able to understand the baseline procedures. The baseline data were collected between 2008 and 2010. Every civil servant, active or retired between the age of 35 and 74 years, was eligible; pregnant women were not included. We excluded those that were not able to understand the instructions of the study, who intended to leave the institution and who did not live in one of the six metropolitan regions [7]. For this study, we also excluded participants with a previous history of stroke or using medication (neuroleptics, anticonvulsants, cholinesterase inhibitors or antiparkinsonian agents) that could indicate the presence of neurologic or psychiatric diseases known to interfere with performance on cognitive tests.

The questionnaires included a wide range of social and biological items, as well as a self-reported morbidity section, that included a question regarding the history of previous stroke. Participants were instructed to bring their prescriptions and packages of any medications they had been using in the previous two weeks [13].

All tests were applied by a trained and certified interviewer, always in the same order and in a quiet environment, with good lighting and low levels of noise or other distractors. Sessions were recorded and then regularly supervised [8].

The Brazilian version of the Consortium to Establish a Registry for Alzheimer’s disease (CERAD’s) was used to test memory and language. The Word Memory Test (WMT) comprises a 10-word list used to evaluate: 1) short-term memory, learning after three trials; 2) delayed recall memory, recalling the word list after five minutes doing another task, and 3) recognition, when the ten words previously presented are mixed to ten new ones. The short-term memory score was obtained by the sum of the words recalled in the three trials, with a maximum score of 30 points. Delayed recall memory score was the sum of all words recalled after five minutes, with a maximum score of 10 points. Recognition score was obtained by the difference between the correct and incorrect words recalled, with a maximum of 10 points [8].

Verbal fluency tests (VFT) included a category test (animals) and a phonemic test (words beginning with the letter F). The supervisors of the six research centers used the same written manual to classify individuals’ responses and mistakes such as intrusions (inappropriate words for the given category or word), perseverations (same word repeated) and general category errors (when a participant mixed general and specific terms, e.g. bird and nightingale).

The high consistency of VFT scores among the six research centers confirmed the reliability of these tests after quality control procedures [14].

The version A of the Trail Making Test B was used as training for the version B (Trail B), as pre-tests revealed that many participants had difficulty in understanding this task. The amount of time spent to complete the task was computed only for the latter. Participants were instructed to complete each version as quickly and accurately as possible, without taking out the pencil from the test sheet. When an error was made, the participant was instructed to return to the circle where the error originated and then to continue the task. In order to avoid participant embarrassment, they were allowed to complete the Trail B, regardless of time; but data were considered only for those participants who performed the test within five minutes [8].

Age was analyzed as a continuous variable and participants were also categorized in three age groups: adults (35–44 years-old), middle-aged (45–64 years-old) and elderly (65–74 years-old). Education level was defined according to the international standard classification of education [12] and combined into four groups: incomplete elementary education (<8 years), complete elementary education and incomplete secondary education (8–10 years), complete secondary education and incomplete tertiary education (11–14 years), tertiary education (>14 years) [15].

### Statistical analysis

Data were analyzed using the statistical software STATA version 11.0 [16]. The medium and interquartile ranges of the distributions of the final score in each test were compared by age group and schooling level among men and women. Multivariable linear regression analysis was used to determine the influence of socio-demographic characteristics (sex, age and schooling) on the distribution of the final score of each cognitive test. The beta coefficients and their confidence intervals, as well as the coefficient of determination, were analyzed in order to understand the magnitude of the influence of each covariate. The possibility of interaction among the independent variables was investigated by stratifying the linear regression analysis by sex and by age [17]. The possibility of using parametric tests was certified by verification of skewness and kurtosis distribution curves for all the cognitive tests (data not shown).

The response rates of cognitive tests were carried out according to age and schooling. The chi-square test was used to compare the differences between proportions. Trends in the frequency of response rate between age-groups were assessed using Maentel-Haenzel chi-square test [17].

### Ethics

ELSA-Brasil was approved by the Research Ethics Committee of all the six participating centers. All the participants signed a Free Informed Consent Term before the collection of clinical and laboratorial data. All international rules regarding data confidentiality in storage and analysis are being followed [7].

## Results

From the 15,105 participants, 14,594 participants remained in the analysis. We excluded 511 participants: 181 who reported a previous stroke and 330 were using medications know for potentially interfering with cognition. Compared to the studied population, these participants were older (11.9 % of adults, 68.5 % of middle-aged and 19.6 % of elderly, *p* = 0.001), more frequently women (59.1 % *p* = 0.035) and slightly less educated (50.3 %, 33.9 %, 7.2 % and 8.6 % with 14+, 11–14, 810, and less than 8 complete years of schooling, respectively; *p* = 0.06). As expected, dropouts presented significantly (*p* < 0.001) lower median scores to immediate memory (20.0 vs. 21 words), categorical VFT (17 vs. 18 words), phonemic VFT (11 vs. 13 words) as well as a higher mean time to perform Trail B (122.8” vs. 109.6”) (data not shown).

Women represent 7916 (54.2 %) of the population; 11,777 (80.7 %) were active workers and 2817 (19.3 %) were retired. Median age was 51 years (mean = 51.97 + 9.05 years). There were 3279 (22.5 %) adults, 9823 (67.3 %) middle-aged and 1492 (10.2 %) elderly. The majority of the population (7693, 52.7 %) had tertiary education, 5060 (34.7 %) had 11 to 14 years of schooling, 991 (6.8 %) had 8 to 10 years of schooling, and 850 (5.8 %) had less than 8 complete years of schooling. Only 21 (0.14 %) participants were illiterate out of 462 (3.16 %) who reported having less than 4 years of schooling.

The WMT was administered to 14,454 (99.0 %) participants, as 140 participants, including the 21 illiterates, were not able to perform the test. Concerning the VFT, 14,568 (99.8 %) participants performed the categorical test and 14,539 (99.6 %) the phonemic test. Trail B was performed by 13,160 (90.2 %) participants who were able to complete the task in up to five minutes.

The score distribution of all cognitive tests is slightly different by sex, with a greater and steady reduction on memory tests and VFT scores, as well as a longer time to perform Trail B, with increasing age and decreasing levels of education (Tables 1 and 2). These associations were observed in crude and multivariable regression analyses. As the results of multivariable analysis using age as a continuous variable were similar to those using age categories we are showing the latter results, since we assumed that cognition decreases differently throughout aging (Table 3).

**Table 1:** Score distribution of memory tests, verbal fluency tests and Trail B by age group and schooling level among male participants of ELSA Brasil, 2008–2010

Median (P_10_–P_90_) of cognitive tests						
Schooling (years)	Memory learning^a^	Recall^b^	Recognition^b^	Verbal fluency animals^c^	Verbal fluency F letter^c^	Trail Making Test B^d^
35–44 years old						
<8	18 (12–24)	6 (2–9)	10 (10–8)	14 (9–18)	8 (5–14)	180 (101–278)
8–10	19 (14–24)	6 (3–8.5)	10 (10–8)	15.5 (10–21)	11 (6–15.5)	116 (98–190)
11–14	21 (17–26)	7 (4–9)	10 (10–9)	18 (12–24)	12 (7–17)	94 (59–162)
14+	23 (18–27)	8 (5–10)	10 (10–9)	21 (15–28)	14 (9–19)	70 (49–111)
45–64 years old						
<8	17 (22–12)	5 (2–8)	10 (10–7)	13 (9–19)	7 (2–13)	180 (120–258)
8–10	18 (14–23)	6 (3–8)	10 (10–8)	14 (9–20)	9 (5–14.5)	166 (95–261.5)
11–14	21 (16–26)	6 (4–9)	10 (10–8)	17 (11–23)	11 (6–17)	120 (71–218.5)
14+	22 (17–26)	7 (5–10)	10 (10–9)	20 (14–27)	14 (9–19)	83 (54–134)
65–74 years old						
<8	15 (10–21)	4 (2–6)	10 (10–6)	12 (7–17)	7 (2–12)	209 (123–275)
8–10	17 (12–23)	5 (2–8)	10 (10–7)	14 (9–21)	9 (3–14)	183 (107–280)
11–14	19 (14–23)	6 (3–8)	10 (10–8)	15 (10–21)	10 (6–15)	147.5 (83–245)
14+	20 (15–24)	6 (3–9)	10 (10–8)	18 (13–25)	13 (8–19)	99 (64–152)

^a^Short-term memory score was obtained by the sum of the words recalled in the three trials, with maximum score of 30 points

^b^Maximum score of 10 points

^c^Number of correct words

^d^Time in seconds

**Table 2:** Score distribution of memory tests, verbal fluency tests, and Trail B by age group and schooling among female participants of ELSA Brasil, 2008–2010

	Median (P_10_–P_90_) of cognitive tests					
Schooling (years)	Memory learning^a^	Recall^b^	Recognition	Verbal fluency animals^c^	Verbal fluency F letter^c^	Trail Making Test B^d^
35–44 years old						
<8	16 (14–19)	6 (0–7)	10 (10–9)	13 (7–17)	7 (2–15)	135 (104–288)
8–10	20 (15–24)	7 (4–9)	10 (10–9)	15 (12–21)	11.5 (5–17)	112.5 (77–180)
11–14	22 (17–26)	8 (5–9)	10 (10–9)	18 (12–24)	13 (7–18)	91 (62–168)
14+	23 (19–27)	8 (6–10)	10 (10–9)	21 (16–27)	14 (9–20)	74 (50–115)
45–64 years old						
<8	18 (14–23)	6 (3–8)	10 (10–7)	12 (8–17)	8 (3–14)	200 (121–280)
8–10	19 (14–24)	6 (4–9)	10 (10–8)	14 (9–20)	9 (4–15)	154 (96–257)
11–14	21 (16–26)	7 (4–9)	10 (10–9)	17 (11–23)	12 (6–17)	119 (72–212)
14+	23 (19–27)	8 (6–10)	10 (10–9)	20 (15–26)	14 (9–19)	86 (58–143)
65–74 years old						
<8	17 (10–22)	5 (3–7)	10 (10–6)	12 (7–17)	6 (3–11)	197 (131–291)
8–10	19 (14–24)	6 (3–8)	10 (10–8)	15 (10–18)	8 (5–12)	173 (107–288)
11–14	20 (15–25)	6 (4–9)	10 (10–8)	15 (10–22)	10 (5–14)	152 (85–254)
14+	21 (16–26)	7 (4–9)	10 (10–9)	19 (14–25)	14 (8–19)	107 (73–172)

^a^Immediate memory score was obtained by the sum of the words recalled in the three trials, with maximum score of 30 points

^b^Maximum score of 10 points

^c^Number of correct words

^d^Time in seconds

**Table 3:** Regression analysis of the performance on cognitive tests, by sex, age and level of education among participants of ELSA-Brasil

Variables	Beta (95 % IC) Univariable analysis)	Adjusted R^2^ (univariable analysis)	Beta (95 % IC) Multivariable analysis^e^	Adjusted R^2^ Multivariable analysis
Immediate memory				
Female^a^	1.54 (1.41;1.67)	0.04	1.34 (1.23; 1.45)	0.18
Age (10 years)^d^	−0.89 (−0.96; −0.83)	0.04	−0.75 (−0.82–0.69)	
Age groups^b^				
Middle-aged	−1.02 (−1.17; −087)	0.03	−0.74 (−0.087; −0.60)^f^	
Elderly	−2.61 (−2.86; −2.38)		−2.23 (−2.44; −2.01)	
Schooling level (years)^c^				
8–10	1.59 (1.24;1.94)	0.13	1.40 (1.07; 1.75)	
11–14	3.41 (3.13;3.70)		2.82 (2.54; 3.10)	
14+	5.16 (4.88;5.44)		4.63 (4.36; 4.90)	
Recall				
Female	0.71 (0.65; 0.78)	0.03	0.62 (0.56; 0.68)	0.16
Age (10 years)	−0.47 (−0.51; −0.44)	0.05	−0.41 (0.45; 0.38	
Age groups				
Middle-aged	−0.56 (−0.64; −0.49)	0.04	−0.43 (−0.50; −0.35)	
Elderly	−1.41 (−1.53; −1.29)		−1.32 (−1.35; −1.12)	
Schooling level (years)				
8–10	0.68 (0.50; 0.86)	0.11	0.58 (0.40; 0.75)	
11-14	1.55 (1.40; 1.69)		1.27 (1.09; 1.37)	
14+	2.37 (2.23; 2.52)		2.09 (1.95; 2.22)	
Recognition				
Female	0.16 (0.14; 0.20)	0.009	0.14 (0.11; 0.17)	0.06
Age (10 years)	−0.16 (−0.17; −0.14)	0.02	−0.13 (−0.15; 0.12)	
Age groups				
Middle-aged	−0.14 (−0.18; −0.11)	0.02	−0.10 (−0.13; −0.07)	
Elderly	−0.46 (−0.52; −0.41)		−0.40 (−0.45; −0.34)	
Schooling level (years)				
8–10^c^	0.31 (0.23; 0.40)	0.04	0.28 (0.20; 0.36)	
11–14^c^	0.56 (0.49; 0.63)		0.47 (0.40; 0.53)	
14+^c^	0.74 (0.67–0.81)		0.67 (0.60; 0.73)	
Verbal fluency tests (animals)				
Female^a^	0.41 (0.24;0.58)	0.001	0.569 (−0.10; 0.021)	0.21
Age (10 years)	−1.11 (−1.20; −1.01)	0.02	−0.87 (−0.96; −0.79)	
Age groups				
Middle-aged^b^	−1.70 (−1.91; −1.50)	0.03	−1.12 (−1.31; −0.94)	
Elderly^b^	−2.99 (−3.31; −2.68)		−2.36 (−2.65; −2.07)	
Schooling level (years) l^d^				
8–10^c^	1.57 (1.13; 2.00)	0.19	1.43 (1.00; 1.86)	
11–14^c^	4.07 (3.73; 4.42)		3.65 (3.30; 3.99)	
14+^c^	7.43 (7.09; 7.76)		7.04 (6.71; 7.38)	
Verbal fluency tests (letter F)				
Female	0.45 (0.30; 0.59)	0.003	0.15 (0.21; 0.29)	0.17
Age (10 years)	−0.76 (−0.84; −0.68)	0.02	−0.53 (−0.61; −0.46)	
Age groups				
Middle-aged	−1.07 (−1.24; −0.89)	0.02	−0.59 (−0.76; −0.43)	
Elderly^b^	−2.09 (−2.36; −1.81)		−1.48 (−1.73; −1.22)	
Schooling level (years)				
8–10	1.73 (1.34; 2.11)	0.16	1.63 (1.25; 2.01)	
11–14	4.01 (3.70; 4.32)		3.72 (3.42; 4.03)	
14+	6.25 (5.95; 6.55)		5.99 (5.69; 6.29)	
Trail B				
Female	−0.75 (−2.53; 1.02)	0.001	2.55 (1.05; 4.95)	0.29
Age (10 years)	14.59 (13.62; 15.54)	0.06	14.1 2 (13.33; 14.96)	
Age groups				
Middle-aged	24.70 (22.66; 26.74)	0.05	20.39 (18.60; 22.18)	
Elderly	39.27 (35.88; 42.65)		38.55 (35.60; 41.50)	
Schooling level (years)				
8–10	−27.32 (−33.13; −21.51)	0.24	−23.35 (−29.01; −17.69)	
11–14	−68.23 (−73.1; −63.37)		−60.27 (−65.06; −55.49)	
14+	−103.5 (−108.3; −98.7)		−96.76 (−101.49; −92.95)	

Reference level: ^a^male, ^b^adults (35–54 years old), ^c^less than 4 years of school

Analysis: ^d^Age in years, as continuous variable, ^e^correlation with sex and schooling using age as a continuous variable. ^f^correlation of age strata adjusted by sex and schooling

The regression analysis showed that women had slightly higher scores than men in all memory tests, but took a higher time to perform Trail B. None or minimum differences were seen between sexes on the performance of VFT. We observed a significant reduction on the performance of all tests with increasing age; as well as a better performance with increasing levels of education. For instance, the scores of the word list and VFT decreased at about one word for every 10 years of aging; whereas higher-educated participants scored five words further on the word list test, as well as six or seven further correct words on VFT, when compared to lower-educated participants. Although also significant, the magnitude of these differences is smaller when comparing the scores of the same tests by age strata (Table 3).

By observing the coefficient of determination (R^2^), the univariate analysis showed that the influence of the level of education on the cognitive tests scores varied from 13 to 19 % for WMT and VFT, respectively, to 24 % for Trail B. It was also observed a significant but smaller influence of sex and age; the R-squared varying from 1 % to 5 %. The multivariable regression analysis showed that sociodemographic characteristics can explain only 6 % of the variation on the performance of the recognition test, but up to 29 % of the variation on Trail B performance (Table 3).

Stratified by sex and by age strata, the regression analysis of cognitive performance by level of education did not suggest any interaction between these variables (data not shown).

Table 4 shows the response rates of the tests. We can observe a high response rate among all tests except for Trail B. Although more than 90 % of participants performed the memory tests, we observed a small but significant and steady decrease on the response rates with increasing of age and decreasing of education level. The response rates to VFT were not influenced by age, but decreased with level of education among the middle-aged on both tests; and among elderly on the phonemic VFT. Response rates were lowest regarding Trail B, decreasing from 99 % among the younger and most educated to 28 % for the older and least educated.

**Table 4:** Response rates of cognitive tests by age and schooling among the 14,594 participants of ELSA-Brasil

Age groups/schooling*	Memory tests^a^ 14,454 (99.0 %)	VFT (animal) 14,568 (99.8 %)	VFT (letter F) 14,539 (99.6 %)	Trail B 13,160 (90.2 %)
35–44 years-old				
14+	1919 (100)	1919 (99.9)	1917 (99.9)	1904 (99.2)
11–14	1230 (100)	1228 (99.8)	1224 (99.5)	1186 (96.4)
8–10	97 (100)	96 (99.0)	96 (99.0)	80 (82.4)
<8	32 (96.7)	33 (100)	33 (100)	20 (60.6)
	p** = 0.001	*p* = 0.15	*p* = 0.10	*p* = 0.001
45–64 years-old				
14+	4929 (99.9)	4924 (99.8)	4924 (99.8)	4868 (98.7)
11–14	3512 (99.9)	3508 (99.7)	3505 (99.7)	3124 (88.8)
8–10	729 (98.8)	732 (99.2)	732 (99.2)	506 (68.6)
<8	541 (85.1)	633 (99.5)	618 (97.2)	279 (43.9)
	*p* = 0.001	*p* = 0.01	*p* = 0.001	*p* = 0.001
65–74 years-old				
14+	842 (100)	841 (99.9)	840 (99.8)	808 (96.0)
11–14	313 (100)	313 (100)	313 (100)	234 (74.8)
8–10	154 (98.7)	155 (99.4)	155 (99.4)	83 (53.2)
<8	157 (86.7)	181 (100)	178 (98.3)	50 (27.6)
	*p* = 0.01	*p* = 0.29	*p* = 0.03	*p* = 0.001

^a^Number and proportions of participants who performed the tests

*Memory and Trial B tests presented a *p* < 0.001 for Mantel-Haenzel chi-square for trend across the age strata

**p values for Chi-square tests across levels of educatio

## Discussion

This study confirmed the influence of socio-demographic characteristics on cognitive performance of adults, with similar findings concerning the small influence of sex. However, it also showed that education rather than age was the most impactful factor for performance in all cognitive tests [18, 19].

As cohort studies usually employ neuropsychological tests to determine the cognitive decline over time, it is important that these tests present the smallest possible influence from other variables than age. The level of schooling seems to be an important covariable in the analysis of cognitive decline, mainly in cohort studies in underdeveloped countries that use tests originally designed to evaluate cognition from developed countries populations. The education was also significantly more important than age on the performance on the VTF among Brazilian elderly [18, 19]. An opposite result was observed in normative data for Trail B performed among 911 Canadian adults (18–89 years old), where age, and not education, had a stronger effect on the performance of this test in this population with a higher level of schooling [20].

Level of education can interact with cognition in different ways. An interesting hypothesis concerning the association between age-related cognitive decline and education proposes three different patterns: a) there would be the same degree of cognitive age-related decline within different educational levels (parallelism), b) less age-related cognitive decline within the well-educated (protection) or c) an age-related reduction of the initial advantage exhibited by the young and middle-aged well-educated group (confluence) [21, 22]. In this study, the parallelism phenomenon may be at play for all tests, but the cross-sectional nature of the data on cognitive status limits inference for cognitive decline over time.

Most of the participants of ELSA-Brasil included in this study are middle-aged. Women (54.2 %) are slightly more represented than men. Women outperformed men on verbal learning and verbal fluency tests, and men outperformed women on visuospatial ability. It is likely that sex hormones influence cognitive domains, but our study design does not permit this evaluation [23].

Memory tests that use a list of simple words should represent the capacity to learn, retain and utilize information, which requires the intact functioning of many brain regions, including some especially susceptible to injury or diseases [24]. The exclusion of psychiatric and neurological diseases, as well the younger composition of the ELSA-Brasil population, may explain why memory tests have been less affected than executive function tests (VFT and Trail B) by sociodemographic variables.

The mean number of correct words in category VFT was similar to that of other studies both in developed and under-developed countries [18, 19, 24]. Verbal fluency requires the systematic retrieval of organized information from semantic memory and, apparently, education facilitates this semantic access, widening an individual’s repertoire of words [24]. The magnitude of the influence of education may differ among populations. A normative study for population-based elderly from undeveloped countries observed similar scores, but with a considerable effect of country site, mainly on the performance of category VFT, after accounting for compositional differences in age, education and sex [25].

The strong influence of education on Trail B performance may be related to poor reading habits among the population studied. Another factor to be considered is the challenge posed by the presence of the letter K in the trail, as this letter is not familiar to Portuguese speakers and had even been abolished from the Brazilian alphabet from 1943 to 1990.

Although there was observed a high compliance to the cognitive evaluation, both age and education influenced the response rates to cognitive tests. Oldest and less educated participants were less prone to perform the cognitive tests, especially the Trail B, and this bias may have contributed to overestimate the median scores in these tests, since these losses presumably would have caused worse results on cognitive evaluation.

The strengths of this study rely on its large adult population from a medium income country and on the high quality assurance of measures. A limitation to this cognitive evaluation is the lack of reasoning tests. In developed countries, the lack of intelligence quotient scores may be not considered a serious limitation given the well-known positive association between education and IQ [21, 22]. This situation could not be generalized to countries such as Brazil, where social inequalities influence the level of education, regardless of intelligence. It is important to consider introducing this evaluation in the ELSA-Brasil follow-up.

Level of schooling among studied participants is higher than that of the Brazilian general population. This probably reflects the social selectivity of civil servants. According to the last national survey in 2010 [26], 11.3 % of Brazilian adults (25+ years-old) had a tertiary education and 49.3 % had less than eight years of schooling (IBGE), as opposed to 52.7 % and 5.8 % of ELSA-Brasil participants, respectively. Although these participants do not represent the Brazilian population, the results of the longitudinal evaluation on cognitive decline, mainly among the young adults and middle-aged participants, may result in significant contributions to better understand, prevent or delay cognitive decline.

When investigating other social determinants of low cognitive performance in this population, we showed that early exposure to adverse social and nutritional conditions seems to be also detrimental to cognitive performance, independent of educational achievement. A study with 12,997 participants (35–64 years) of ELSA-Brasil revealed lower maternal education increased the chances of poor performance in all cognitive tests, with a dose-response gradient. Low birth-weight was related to poor performance in Trail B and greater trunk length decreased the chances of poor performance in both verbal fluency tests and Trail B [27].

In order to perform cross-sectional analyses, we are using z-scores standardized to give a normal curve to each cognitive test. To control the effect of age and education on scores, the subjects were stratified into the three age groups (34–44–45–64 and 65+ years) and the four levels of education (<8, 8–10, 11–14 and 14+ years of schooling) we used in this study. Lowered global cognitive function is defined as one or more standard deviation below average in each age and education strata [4, 28]. Besides, as our study aims to perform multiple cognitive tests over time, we will be able to investigate the direction and magnitude of the influence of education on aging cognitive decline of this cohort of workers from a developing country.

## Conclusions

This article adds information on the levels of cognition of a large and heterogeneous Brazilian adult population and highlights the importance of considering education when studying cognitive status in settings where deep social inequalities are present. Our results indicate that education influences cognition much more than age in this specific population. These findings reinforce the need for caution in analyzing and diagnosing cognitive impairments based on traditional cognitive tests cutoffs and the importance of searching for cognitive tests that are not affected by education, in order to properly study the less educated population, presumably at higher risk to cardiovascular diseases and cognitive decline.

## Abbreviations

ELSA-Brasil: Estudo Longitudinal de Saúde do Adulto (Longitudinal Study of Adult Health)
IQ: Intelligence quotients
Trail B: Trail making test, version B
VFT: Verbal fluency tests
WMT: Word memory tests
